# An Algorithmic Approach for Assessment of Mediastinal Lesions Using Conventional Transbronchial Needle Aspiration and Endoscopic Ultrasonography in a Single Procedure

**DOI:** 10.1155/2017/1971629

**Published:** 2017-06-14

**Authors:** Andrew Burkett, Harmanjatinder S. Sekhon, Craig Burkett, Shaheed W. Hakim, Kayvan Amjadi

**Affiliations:** ^1^Division of Respiratory Medicine, The Ottawa Hospital, University of Ottawa, Ottawa, ON, Canada; ^2^Department of Pathology and Laboratory Medicine, The Ottawa Hospital, University of Ottawa, Ottawa, ON, Canada; ^3^Department of Statistical Sciences, University of Toronto, Toronto, ON, Canada

## Abstract

**Background:**

In the era of endobronchial/esophageal ultrasound (EBUS-TBNA/EUS-FNA), many centers forgo conventional transbronchial needle aspiration (C-TBNA) in favour of EBUS-TBNA/EUS-FNA despite no conclusive evidence showing better yields with EBUS-TBNA/EUS-FNA.

**Objectives:**

Assess the feasibility of an algorithmic approach for mediastinal sampling beginning with C-TBNA utilizing rapid onsite cytologic evaluation.

**Methods:**

Descriptive analysis of 92 consecutive patients referred for adenopathy that underwent C-TBNA and subsequent EBUS-TBNA/EUS-FNA if C-TBNA was negative or nondiagnostic.

**Results:**

92 procedures were analyzed. In 50 (54.3%) of cases, C-TBNA alone was sufficient. EBUS-TBNA was performed after C-TBNA in 27 (29.3%) of cases and EUS-FNA in 33 (35.9%) of cases. The yield was 92.9% for C-TBNA, 92.5% for EBUS-TBNA, and 89.7% for EUS-FNA. There were no statistically significant differences in yields by LN station (*P* = 0.51), the relationship between yield and LN size (*P* = 0.37), or time difference in procedures following the algorithm compared to EBUS/EUS only procedures (33.7 minutes versus 32.4 minutes on average [95% CI for difference: −9.1 to 11.7], *P* = 0.80).

**Conclusions:**

An algorithmic approach to assess the mediastinum using C-TBNA initially is feasible without sacrificing yield or procedure times. C-TBNA was sufficient for diagnosis in 54.3% of cases and can be efficiently taught in an IP training program.

## 1. Introduction

Tissue sampling of mediastinal lymph nodes (LNs) is often required during assessment of patients with mediastinal lesions or for accurate staging of patients with lung cancer. Endoscopic modalities are safe and minimally invasive tools with excellent diagnostic yield [1, 2]. Conventional transbronchial needle aspiration (C-TBNA) can be performed during routine bronchoscopy. However, its diagnostic yield in assessment of mediastinal and hilar LN in patients with lung cancer is approximately 80% [3]. The sensitivity of C-TBNA depends heavily on radiographic size of the targeted LN [4], anatomical location of the targeted LN [5], number of needle aspirates [6], availability of rapid onsite evaluation (ROSE) [6], experience of the operator [7], and the study population [8].

Despite its reported safety and high impact on patient management [9], surveys indicate that C-TBNA is used by a mere 10–30% of pulmonologists on a routine basis [10, 11]. The main reasons for not utilizing C-TBNA was the belief that C-TBNA was not useful or that the performing pulmonologist was not confident in their C-TBNA bronchoscopic technique [10]. Furthermore, physicians are uncomfortable with the lack of real-time visualization of the LN and biopsy needle [7]. Introduction of endoscopic ultrasonography has provided a solution for the latter problem.

Endobronchial ultrasound-guided TBNA (EBUS-TBNA) provides real-time visualization and access to paratracheal, subcarinal, and hilar LN [2]. Transesophageal ultrasound-guided fine needle aspiration (EUS-FNA) provides physicians with real-time access to LN located adjacent to the esophagus [12]. As a result, EBUS-TBNA and EUS-TBNA can be employed as complimentary modalities providing endoscopists with theoretical access to the entire mediastinal and hilar regions [12]. In fact, recent studies have suggested that the combined procedures appear to improve the accuracy of mediastinal staging (compared to either procedure alone) resulting in diagnostic yields greater than 90% [13, 14]. Generally, EBUS-TBNA and EUS-TBNA are performed using two separate devices, by two distinctive physicians (i.e., a pulmonologist and a gastroenterologist), on two different days, thus, limiting the clinical utility of the combined approach. More recently, the two procedures have been performed in the same setting successfully, using an EBUS bronchoscope alone [12, 15]. Although there are several limitations in adopting such approach, its convenience makes it clinically appealing in the hands of trained bronchoscopists.

The current guidelines now specifically recommend EBUS or EUS as the initial diagnostic modality for minimal invasive assessment of the mediastinum [16] but, prior to 2013, the guidelines were more vague suggesting either C-TBNA or EBUS [17]. As our hospital is a training center, utilizing C-TBNA and EBUS in an algorithmic approach allowed us to teach both techniques without compromising patient care. According to the authors from one study, sequential use of various modalities starting with C-TBNA, followed by ultrasound-guided modalities in nondiagnostic cases, followed by surgical exploration reserved for those who remained nondiagnostic after the 2 former procedures, would result in the most cost-effective approach to mediastinal staging among patients with lung cancer [18]. One may question the clinical utility of this sequential approach, as it may result in diagnostic delays and the need for multiple procedures for patients. At our center, we previously used an algorithmic approach utilizing C-TBNA as an initial diagnostic modality followed by EBUS-TBNA and EUS-TBNA, if necessary, performed during the same endoscopic evaluation, for cases where C-TBNA fails to provide a diagnosis after assessment with rapid onsite cytologic evaluation (ROSE). In this paper, we present our experience using this algorithm in a teaching center and describe patient outcomes among 92 consecutive patients who were treated according to our algorithm.

## 2. Study Design

### 2.1. Patients

**Figure 1:**
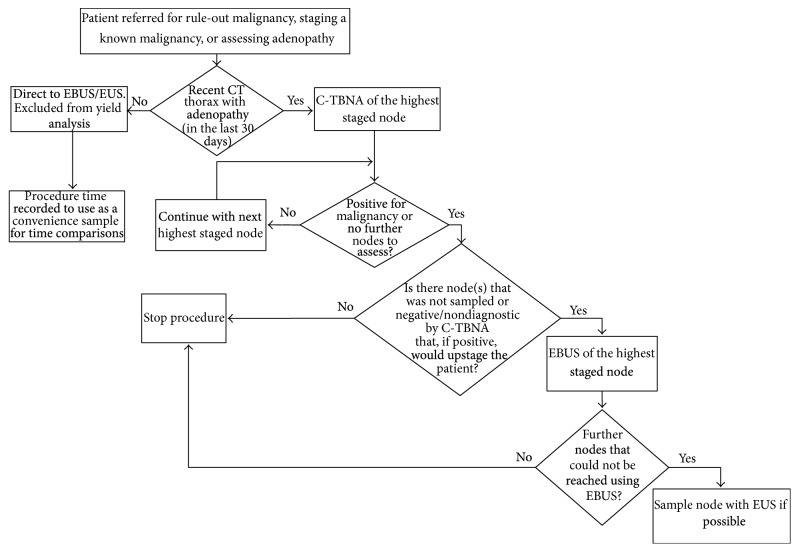
Flowchart describing endoscopy assessment algorithm.

The Ottawa Hospital Research Ethics Board provided approval for this study (20130521-01H). A retrospective analysis of 100 consecutive patients who were 18 years or older and referred for assessment of mediastinal adenopathy from October 2012 to September 2013 was performed. These patients had been examined with a stepwise approach as follows: patients with a computed tomography (CT) scan in the last 30 days showing any visible adenopathy (LN measuring greater than 4 mm in axial or coronal slices) were first assessed by regular bronchoscopy. C-TBNA biopsies of the highest abnormal node that, if positive, would upstage any diagnosis of malignancy were attempted (most commonly station 2, 4, or 11 contralateral to any lung lesion which would stage N3 disease) and reviewed by ROSE. If this resulted in a diagnosis, further LN passes were done to obtain tissue for cell block analysis and the procedure was then aborted. If no diagnosis was made, C-TBNA was attempted on the next highest staged LN group that appeared abnormal (typically station 7 or ipsilateral 4 which would stage N2 disease) and this process would repeat until there were no further abnormal LN groups to assess. If all LN groups were negative and there was an endobronchial lesion or mass on CT, endobronchial biopsies, bronchoalveolar lavage, or transbronchial biopsies were performed as indicated. After assessment with regular bronchoscopy, EBUS was performed if there remained LNs that were either negative for malignancy by C-TBNA or not sampled with C-TBNA due to anatomical location that, if positive for malignancy, would impact staging. If further nodes existed that were not reachable by EBUS or the endoscopist felt were easier to reach with EUS, EUS was finally performed. Patients in this time frame who did not have a CT scan in the last 30 days were assessed only using EBUS/EUS and were excluded from the statistical analysis for yield and used as a convenience sample for procedure time comparison (Figure 1).

Data were collected on the patient's basic demographics and reason for referral. During the procedure, the endoscopy nurse recorded the start and end time of the total procedure, the start and end time of the regular bronchoscopy, the start and end time of the EBUS bronchoscopy, whether an Interventional Pulmonology (IP) fellow or IP staff physician was doing the case, and number of passes for each LN group. Data were also collected on size of LN on axial CT images, final pathological diagnosis for each biopsy specimen, and number of cells in the cell block. For each biopsy specimen, adequacy of sample size to perform ancillary studies was documented on pathology review.

## 3. Methods

### 3.1. Lymph Node Sampling

Samples from C-TBNA, EBUS-TBNA, or EUS-FNA were considered “diagnostic” if specific cytomorphological abnormalities were identified by a pathologist, “indeterminate” if the pathologist identified abnormal cells but could not make a diagnosis, “negative” if normal lymphocytes were obtained, and “nonrepresentative” if no lymphocytes or abnormal cells could be identified. Diagnostic, indeterminate, or negative samples were considered positive yield while nonrepresentative samples were considered negative yield. Benign samples were followed by either surgical staging or minimum of 1 year radiological follow-up.

Biopsy specimen adequacy was only analyzed if a sample was positive for malignancy. This was conducted by two independent pathologists and any discordance in cell count was reviewed until they reached an agreement. Samples were deemed “inadequate” if they had less than 10 cells, “borderline” if they had 10–49 cells, “acceptable” if they had 50–199 cells, or “ideal” if they had 200 or more cells in the cell block [19, 20].

#### 3.1.1. Endoscopists

All procedures were performed by an attending IP physician or by an IP fellow under direct supervision by their IP attending. There were 2 IP fellows performing endoscopy procedures during that period, each in their 6th year of clinical training and both with over 2 years of experience in performing C-TBNA and EBUS/EUS.

#### 3.1.2. C-TBNA

C-TBNA was performed using an Olympus T-190 bronchoscope with a 2.8 mm working channel. 22-gauge needles were used for all samples. The “jabbing method” was used for all biopsies.

#### 3.1.3. EBUS-TBNA

EBUS-TBNA was performed through an Olympus UC180F bronchoscope with a 2.8 mm working channel using an EMU1 ultrasound processor. Target LNs were identified and samples were taken from LN using standard methodology [2].

#### 3.1.4. ROSE

Cytology specimens were air-dried on site and stained by a ROSE cytotechnologist who then gave an opinion on whether a representative sample was obtained. After this, 2–5 more passes in the same location were done to obtain tissue for cell block analysis.

### 3.2. Outcomes

Our outcomes were the diagnostic yields of C-TBNA, EBUS-TBNA, and EUS-FNA, diagnostic yield as a function of LN station, diagnostic yield as a function of LN size, the percentage of cases where additional ultrasonographic procedures were required after C-TBNA, average number of cells per LN aspirate, and proportion of samples with sufficient cells to perform ancillary studies if needed.

## 4. Analysis

Continuous variables are presented as means and standard deviations and binary variables as numbers and percentages. Two sample* t*-tests were used for continuous variables and Pearson's chi-square test or Fisher's exact test was used for counts as appropriate. Binary outcomes were analyzed with the use of logistic regression models. Two-sided *P* values of less than 0.05 were considered to indicate statistical significance. The analysis was performed using* R* Statistical Software, v2.15.1.

## 5. Results

### 5.1. Patients

The first 100 patients referred for assessment for adenopathy were screened. Eight patients did not have a CT in the preceding month and so went directly to EBUS/EUS and were excluded from yield calculations. The remaining 92 all met the inclusion criteria and therefore were enrolled. All 92 patients were included in the final analysis and 206 LN stations were sampled.  Table 1 describes the procedural conditions and demographics of the patients enrolled in the study.

### 5.2. Outcomes

#### 5.2.1. Diagnostic Modality

**Figure 2:**
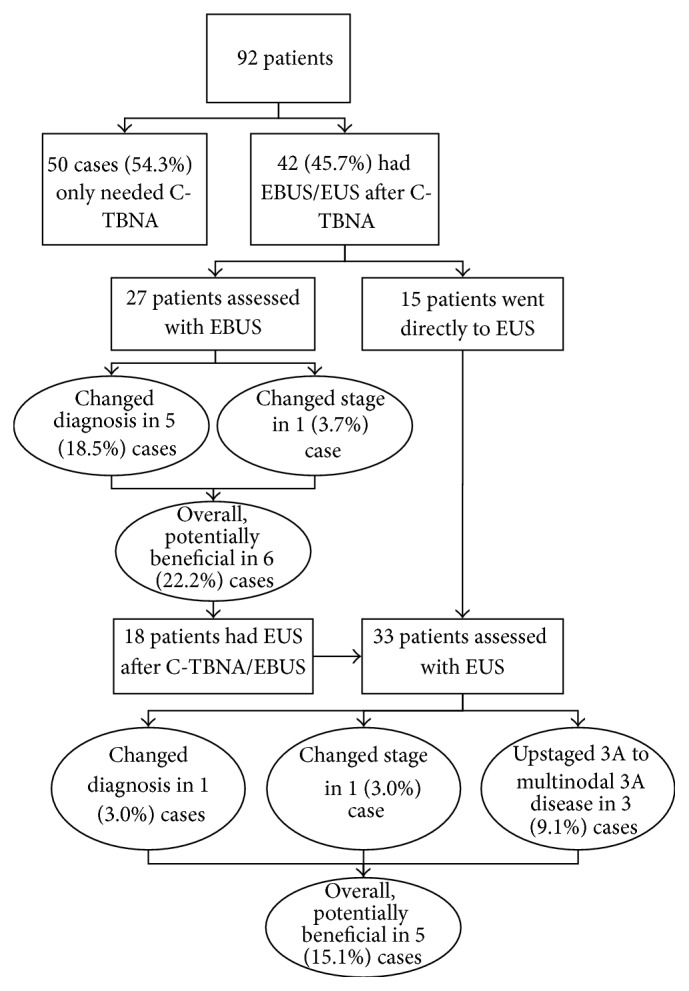
Diagnostic utility of each algorithm arm.

92 procedures were analyzed and summarized in Figure 2. C-TBNA alone was sufficient in 50 (54.3%) of the cases. For the remaining 42 (45.7%) cases, EBUS-TBNA, EUS-FNA, or both were performed after C-TBNA.

#### 5.2.2. EBUS Utility after C-TBNA

In 27 cases, C-TBNA was followed by EBUS. EBUS results potentially changed management by providing a diagnosis in 5 (18.5%) cases and upstaged a previously made diagnosis in 1 (3.7%) case. Overall, utilizing EBUS after C-TBNA was potentially beneficial in 6 (22.2%) of cases.

#### 5.2.3. EUS Utility after C-TBNA or EBUS

In 33 cases, EUS was performed after either C-TBNA or EBUS. EUS potentially changed management by providing a diagnosis in 1 (3.0%) case and upstaged a previously made diagnosis in 1 (3.0%) case. In a further 3 (9.1%) of cases, EUS upstaged 3A to multinodal 3A disease which may affect treatment decisions. Overall, utilizing EUS after C-TBNA or EBUS was potentially beneficial in 5 (15.1%) of cases.

#### 5.2.4. Yield

C-TBNA was done on 127 LN groups; 72 of the samples were diagnostic (56.7%), 12 (9.4%) were indeterminate, 34 were negative (26.8%), and 9 (7.1%) were nonrepresentative demonstrating a 92.9% yield for C-TBNA overall. EBUS-TBNA was performed on 40 LN groups; 12 of these samples were diagnostic (30.0%), 1 (2.5%) was indeterminate, 22 were negative (55.0%), and 2 (5.0%) were nonrepresentative demonstrating a 92.5% yield for EBUS-TBNA overall. EUS-FNA was performed on 39 LN groups; 15 of these samples were diagnostic (38.5%), 2 (5.1%) were indeterminate, 23 were negative (59.0%), and 2 (5.1%) were nonrepresentative demonstrating an 89.7% yield for EUS-FNA overall.

The difference in yield between C-TBNA biopsies performed by an IP fellow compared to a staff physician was not statistically significant (93.0% versus 92.6%, *P* = 1.00).

#### 5.2.5. Lymph Node Location

The difference in yields by LN station overall and in C-TBNA, EBUS-TBNA, and EUS-FNA is shown in Table 2. Logistic regression models did not show a statistically significant difference in yields by LN location overall (*P* = 0.51), C-TBNA alone (*P* = 0.19), EBUS-TBNA alone (*P* = 0.42), or EUS-FNA alone (*P* = 0.73).

#### 5.2.6. Lymph Node Size

Logistic regression models did not show a statistically significant relationship between yield and LN size overall (*P* = 0.37). Furthermore, the relationship between yield and LN size was not affected by biopsy modality (*P* = 0.88).

#### 5.2.7. Procedure Time

There was no significant difference in procedure times between cases starting with C-TBNA and following the algorithm and those using EBUS/EUS-TBNA only (33.7 minutes versus 32.4 minutes on average [95% CI for difference: −9.1 to 11.7], *P* = 0.80). There was no statistically significant difference in procedure times if a fellow did the procedure compared to a staff physician (average total length of procedure 33.9 minutes for fellows versus 33.1 minutes for staff physicians [95% CI for difference: −3.9 to 4.9], *P* = 0.83).

#### 5.2.8. Ancillary Studies

The average number of cells per LN sampled was 458 cells using C-TBNA (147 cells per pass), 693 cells using EBUS-TBNA (205 cells per pass), and 631 cells using EUS-FNA (217 cells per pass). 87.3% of samples obtained using C-TBNA had an adequate number of cells to perform ancillary studies if needed compared to 85.7% of EBUS-TBNA and EUS-FNA samples (*P* = 1.00). 57.1% of samples obtained using C-TBNA had an ideal number of cells to perform ancillary studies if needed compared to 52.4% of EBUS-TBNA and EUS-FNA samples (*P* = 0.70).

## 6. Discussion

Lung cancer is currently the number one cause of death from malignancy in both men and women and the incidence of lung cancer is increasing mostly due to increased rates among females even though rates in males are decreasing slightly [21]. This fact, combined with the increasing availability of CT scanning, has led to an increasing number of referrals for mediastinal staging for a suspected or confirmed lung cancer. In addition, many centers worldwide are currently considering offering CT screening for high risk patients [22] which may lead to a dramatic increase in referrals for mediastinal staging. Strategies for quickly and accurately assessing these patients while minimizing costs are needed.

This study demonstrates the feasibility of using an algorithmic approach for assessment of mediastinal lesions in a teaching center where there is access to ROSE. This is important for multiple reasons: cost, patient safety, and training pulmonologists who may not have access to endoscopic ultrasound modalities in their future place of employment.

Surprisingly, in this analysis, the average procedure times for cases beginning with C-TBNA and moving to EBUS/EUS if necessary were similar to those using EBUS only. It is important, however, to note that patients were not randomized and, as patients with a preexisting diagnosis of malignancy were usually rapidly referred for staging (and would present with a recent CT), this may be an unfair comparison to the EBUS group as they could represent a group with a higher proportion of benign disease. However, it is reassuring to note that the algorithmic approach does not appear to increase procedure time.

Many graduating pulmonologists will practice in centers where EBUS is not readily available and those pulmonologists should feel comfortable safely and accurately staging the mediastinum with C-TBNA. This study also demonstrates the feasibility of safely teaching C-TBNA as part of an algorithmic approach in a teaching center without lengthening procedure times or sacrificing yield and adds to recent reports that yield is maintained with C-TBNA compared to EBUS when C-TBNA is performed by experienced bronchoscopists [23].

Interestingly, although the mean number of cells obtained per pass using EBUS was greater than that obtained per pass using C-TBNA, a large amount of tissue was still obtained by C-TBNA. In addition, there were no significant differences in rates of obtaining samples with either an adequate or ideal number of cells between the C-TBNA and EBUS groups. This is reassuring for pulmonologists who are currently using C-TBNA.

This study was descriptive in nature and it lacks a control group of cases evaluated without use of the algorithm for comparisons sake. Despite this limitation, we believe that following an algorithmic approach enhances the ability to teach IP fellows C-TBNA techniques without compromising yield. Now that current guidelines specifically recommend EBUS or EUS as the first step for minimally invasive mediastinal staging [16], further research directions should include a prospective cost-benefit analysis of this algorithm compared to the recommended EBUS/EUS approach. The limited training by pulmonologists in EUS also limits the generalizability in this study regarding the results from EUS.

In conclusion, an algorithmic approach to assessing the mediastinum using C-TBNA initially is feasible without sacrificing yield or procedure times when utilizing ROSE. C-TBNA was sufficient for diagnosis of mediastinal adenopathy in 54.3% of cases and can be safely taught in an IP training program. EBUS-TBNA or EUS-FNA should be reserved and used in a complimentary role to assess nondiagnostic results obtained using C-TBNA.

## Figures and Tables

**Table 1:** Characteristics of patients referred for assessment of mediastinal lesions.

Patients	(*n* = 92)	%
Referred for		
Assessing adenopathy	12	13.0%
Rule-out malignancy	80	87.0%
Male	49	53.3%
Median age	65.2	(32–88)
IP fellow present		69.6%
Final diagnosis		
Malignant		
Adenocarcinoma	31	33.7%
Squamous cell carcinoma	17	18.48%
Small cell lung cancer	14	15.22%
Poorly differentiated	9	9.78%
Metastatic breast cancer	1	1.09%
Metastatic RCC	1	1.09%
Sarcoma	1	1.09%
Benign		
Negative for malignancy	12	13.04%
Sarcoid	5	5.43%
Other benign disease	1	1.09%

**Table 2:** Yield by lymph node location and biopsy modality.

LN	Positive yield							
	C-TBNA		EBUS after C-TBNA		EUS after C-TBNA		Overall	
2R	6/6	100.00%	3/3	100.00%	1/1	100.00%	10/10	100.00%
4R	42/45	93.33%	9/11	81.82%	0/0		51/56	91.07%
4L	9/9	100.00%	1/1	100.00%	13/14	92.86%	23/24	95.83%
6	0/0		0/0		0/0		0/0	
7	38/39	97.44%	8/9	88.89%	5/6	83.33%	51/54	94.44%
8R	2/2	100.00%	0/0		12/14	85.71%	14/16	87.50%
8L	0/0		0/0		4/4	100.00%	4/4	100.00%
10L	2/2	100.00%	0/0		0/0		2/2	100.00%
11R	11/15	73.33%	11/11	100.00%	0/0		22/26	84.62%
11L	8/9	88.89%	5/5	100.00%	0/0		13/14	92.86%

Total	118/127	92.91%	37/40	92.50%	35/39	89.74%	190/206	92.23%

## Abbreviations

C-TBNA: Conventional transbronchial needle aspiration
EBUS: Endobronchial ultrasound
EBUS-TBNA: Endobronchial ultrasound transbronchial needle aspiration
EUS-FNA: Esophageal ultrasound fine needle aspiration
EUS: Esophageal ultrasound
IP: Interventional Pulmonology
LN: Lymph node
ROSE: Rapid onsite cytologic evaluation.
